# Beneficial coinfection can promote within-host viral diversity

**DOI:** 10.1093/ve/vey028

**Published:** 2018-10-01

**Authors:** Asher Leeks, Ernesto A Segredo-Otero, Rafael Sanjuán, Stuart A West

**Affiliations:** 1 Department of Zoology, University of Oxford, Oxford, UK; 2 Institute for Integrative Systems Biology (I2SysBio), Universitat de València, València, Spain

**Keywords:** phenotype mixing, diversity, evolution, multipartite, frequency dependence, coinfection

## Abstract

In many viral infections, a large number of different genetic variants can coexist within a host, leading to more virulent infections that are better able to evolve antiviral resistance and adapt to new hosts. But how is this diversity maintained? Why do faster-growing variants not outcompete slower-growing variants, and erode this diversity? One hypothesis is if there are mutually beneficial interactions between variants, with host cells infected by multiple different viral genomes producing more, or more effective, virions. We modelled this hypothesis with both mathematical models and simulations, and found that moderate levels of beneficial coinfection can maintain high levels of coexistence, even when coinfection is relatively rare, and when there are significant fitness differences between competing variants. Rare variants are more likely to be coinfecting with a different variant, and hence beneficial coinfection increases the relative fitness of rare variants through negative frequency dependence, and maintains diversity. We further find that coexisting variants sometimes reach unequal frequencies, depending on the extent to which different variants benefit from coinfection, and the ratio of variants which leads to the most productive infected cells. These factors could help drive the evolution of defective interfering particles, and help to explain why the different segments of multipartite viruses persist at different equilibrium frequencies.

## 1. Introduction

Viruses can form exceptionally diverse populations inside hosts, with thousands of distinct genetic variants infecting a single host (Lauring and Andino 2010). Infections with a high viral diversity can be more virulent, for example, by infecting more tissue types or through reaching higher viral titres (Vignuzzi et al. 2006; Coffey et al. 2011; Shirogane, Watanabe, and Yanagi 2012; Cao et al. 2014; Bordería et al. 2015; Skums, Bunimovich, and Khudyakov 2015; Xue et al. 2016). Infection diversity also influences virus evolution, as more diverse populations may develop antiviral resistance more rapidly, may be more likely to adapt to new hosts, and can recombine, leading to the emergence of novel pathogens (Bonhoeffer et al. 1997; Bordería, Stapleford, and Vignuzzi, 2011; Ke et al. 2015; Pérez-Losada et al. 2015). The existence of high within-host diversity presents an evolutionary problem, because different variants of the same virus compete to infect a limited population of host cells (Gause 1934; Clarke et al. 1994; Moya et al. 2000). Consequently, why do faster-replicating variants not out-compete slower-replicating variants, leading to a loss of variant diversity?

Several mechanisms have been suggested to promote variant coexistence. If different variants specialise on infecting different cell types, then this could reduce competition, allowing variants to coexist (Elena, Miralles, and Moya 1997; Yuste, Moya, and López-Galı´ndez 2002; Wilke, Reissig, and Novella 2004; Arbiza, Mirazo, and Fort 2010). Alternatively, if the mutation rate is high enough, a diverse set of variants could be maintained through mutation-selection balance (Wilke 2005; Domingo, Sheldon, and Perales 2012; Andino and Domingo 2015). The relative importance of these hypotheses depends upon the extent to which different variants do infect different tissues, and whether the mutation rate is high enough, respectively. Another possibility to explain variant coexistence is if cells infected by multiple viral variants produce more virions, or more effective virions, than singly infected cells (Roossinck, Sleat, and Palukaitis 1992; Qiu and Scholthof 2001; López-Ferber et al. 2003; Shirogane, Watanabe, and Yanagi 2012; Andino and Domingo 2015; Xue et al. 2016; Díaz-Muñoz, Sanjuán, and West 2017; Hisano et al. 2018). This ‘beneficial coinfection’ hypothesis could work via viruses sharing gene products when infecting the same cell, resulting in phenotype mixing (Novella, Reissig, and Wilke 2004; Závada 1976). For example, if multiple mutations are beneficial, but result in different changes to the same gene, then they may not be compatible in the same genome. Therefore, variants with different beneficial mutations could mix in a synergistic way.

However, the viability of the beneficial coinfection hypothesis is not clear. Beneficial coinfection might just slow down the extinction of less fit variants by ‘masking’ fitness differences, rather than allow the long-term coexistence of different variants (Godfray, O'Reilly, and Briggs 1997; Wilke and Novella 2003; Froissart et al. 2004; Wilke, Reissig, and Novella 2004; Gao and Feldman 2009; Loverdo and Lloyd-Smith 2013). Alternatively, this hypothesis might require unrealistically high rates of coinfection, or unrealistically large benefits of coinfection, in order to allow variants to coexist. Could population bottlenecks, a common feature of virus life cycles, reduce the extent to which different variants can interact beneficially (Zwart and Elena 2015; McCrone and Lauring 2018)? Finally, if different variants benefitted differently from coinfection, then the variant which benefitted the most could be favoured disproportionately, reducing coexistence.

We investigated the theoretical plausibility of the beneficial coinfection hypothesis. Our specific aims were to: (1) test how frequent and how beneficial coinfection needs to be for a slower-replicating variant to coexist at equilibrium with a faster-replicating variant; (2) test how bottlenecks in the virus population affect coexistence; (3) investigate the effect of asymmetries in how variants benefit from and contribute to beneficial coinfection. We use an equilibrium modelling approach based on population genetics which we attempt to parameterise using real data. We then follow this up with more realistic simulations of virus growth in cell culture.

## 2. Equilibrium model

### 2.1 Model overview

We have deliberately kept our model as simple as possible, to capture the possible role of beneficial coinfection in a manner that does not depend upon the biological details of certain viruses. For example, we do not model a specific mechanism for coinfection benefit, since this would require making assumptions based on a particular system. Instead, we choose parameters which could result from many different specific mechanisms for coinfection benefit.

We assume that two variants of a virus, A and B, infect the same kinds of cells inside a host. We assume that the rate of spread of each variant within the host depends on the number of virions each variant produces in a given amount of time, as well as the chance that these virions successfully infect further host cells (Klasse 2015). Fitness differences between the variants could therefore stem from mutations which: increase the total number of virions released from an infected host cell; increase the speed of the infection cycle; or which increase the effectiveness of the virions produced. In order to avoid making specific assumptions about the nature of fitness differences between the variants, we capture these factors in a single parameter, ‘productivity’. The productivity of a focal host cell is the relative number of further host cells which are successfully infected by virions produced in the focal host cell in a given amount of time; therefore productivity is analogous to the basic reproductive ratio (*R*_0_) at a cellular level (Bonhoeffer et al. 1997; Nowak et al. 1997). We can express the rate of spread of each variant within the host in terms of its share of the productivity of the cells it infects. This method is formally analogous to treating the two variants as different alleles at a locus in a population genetics model, where phenotypes are infected host cells, alleles are the different viral variants, genotypes are the combinations of viral variants infecting each host cell, the ploidy is specified by the likelihoods of different multiplicities of infection, and fitness is productivity of infected host cells (Chao 1991; Wilke 2005; Otto and Day 2007; Elena et al. 2011).

### 2.2 Lifecycle

We are interested in the maintenance of diversity within a host, and so we model the evolution of an infection inside a single host. We examine the situation when a host is infected by two variants, and ask when this will lead to coexistence, or to one variant outcompeting the other. The two variants could arise through both initially coinfecting the host, or by mutation.

**Figure 1.**
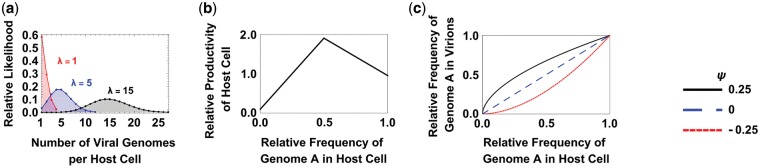
Equilibrium model assumptions. (a) We assume that infection of host cells is well described by a Poisson distribution (*P*(*λ*)), where the Poisson parameter *λ* is given by the ratio of virions to susceptible host cells (MOI). We truncate our Poisson distribution at 1, to focus on infected cells, and at three standard deviations above the mean, to avoid a potentially infinite number of infection states. (b) We assume mixed infections are more productive. The productivity of an infected host cell (*y*-axis) shows a peaked relationship with relative proportion of variant *A* virions in the initial infection mixture (*p_A_*; *x*-axis) according to the function *Φ*(*p_A_*). (c) We assume that the proportion of A genomes in the virions produced by that cell (*y*-axis) can be either linearly or non-linearly related to the proportion of A genomes that initially infected the host cell (*p_A_*; *x*-axis), according to the function *Π*(*p_A_*).

We assume that virions infect host cells according to a Poisson process where the Poisson parameter ***λ*** represents the ratio of virions to host cells. We assume that host cells and virions are well mixed, and that each virion contains only one viral genome, such that the multiplicity of infection (MOI) is equivalent to the ratio of virions to host cells (***λ***). The relative likelihood of a cell being infected by *k* virions is therefore given by the function *P*(*k*), defined as $\left(e^{-\lambda }\;\frac{\lambda^{k}}{k!})/(\sum_{n=1}^{m}e^{-\lambda }\;\frac{\lambda^{n}}{n!}\right)$, where ***λ*** is the ratio of virions to host cells, *k* is the number of virions infecting each host cell, and *m* is defined as $\lambda +3\sqrt{\lambda }$ and is chosen to ensure that we consider >99 per cent of possible infection states. The numerator of *P*(*k*) gives the likelihood of a cell infected by *k* virions where *k* is a Poisson-distributed discrete random variable around ***λ***, and the denominator is a normalisation factor to ensure that we consider the relative likelihoods of each infection state. For full details, see the Supplementary data. *P*(*k*) is illustrated in Fig. 1a for different MOI values.

We further assume that the likelihood of different variant combinations infecting each cell is described by a Binomial process. To achieve this we use a function Bxt,k,i=kixti(1-xt)k-i, where $x_{t}$ is the relative frequency of variant *A* at time t (and so ${1-x}_{t}$ is the relative frequency of variant *B*), *k* is the number of virions infecting the host cell, and *i* is the number of virions infecting the host cell which are variant *A*. For example, when two virions infect a host cell (*k* = 2), the possible infection outcomes are AA (*i* = 2), AB (*i* = 1), and BB (*i* = 0). The relative likelihoods of these are given by $pr\left(AA\right)=B\left(x_{t},\;2,\;2\right)={x_{t}}^{2};pr\left(AB\right)=B\left(x_{t},\;2,\;1\right)=2\;x_{t}\;\left(1-x_{t}\right);\;pr\left(BB\right)=B\left(x_{t},\;2,\;0\right)={{(1-x}_{t})}^{2}.$

We assume that the productivity of an infected host cell *i* (*W_i_*) depends on the viral genes which are expressed. Therefore, the productivity depends on the viral genomes that initially infect the cell. For example, if a host cell is infected only by genomes of variant *A*, we assume that its productivity is *W_A_*. For consistency, we assume that variant *A* has a higher productivity when in pure infection than variant *B*, and consequently spreads more quickly inside the host, all else being equal (*W_A_ *>* W_B_*). As we are focusing on the effects of coinfection of different variants, we also assume that cells infected by multiple genomes of the same variant have the same productivity as cells infected by just one genome of that variant (*W_A_* or *W_B_*) (Timm and Yin 2012).

When a host cell is infected by a mixture of genomes of both variants, then genes from both variants *A* and *B* can be expressed (Novella, Reissig, and Wilke 2004; Závada 1976). Therefore, the total productivity of the cell could be different from *W_A_* or *W_B_*. We capture beneficial coinfection by allowing these mixed infections to have a higher maximum productivity than the most productive pure infections (*W_M_ *>* W_A_*). Therefore, coinfection benefit could arise from different kinds of biological interactions: host cells infected by both variants may produce more virions, may produce virions that are more effective at infecting new cells, or may have a faster infection cycle.

To determine the productivity of cells infected by different combinations of variants *A* and *B*, we use a discontinuous function where we independently specify the productivities of cells infected only by variant *A*, only by variant *B*, or by a mixture of both variants. This is given by

ϕPA=WB+pAWM-WBτA         0≤pA<τAWM             τA≤ pA≤τBτBWA-WMτB-1+pAWM-WAτB-1  τB< pA≤1

Here, *p_A_* is the relative proportion of variant *A* infecting a given host cell (and is given by *i/k*), *W_A_* and *W_B_* give the productivities of a cell infected entirely by variant *A* or entirely by variant *B*, respectively, *W_M_* gives the maximum possible productivity of a cell infected by both variants, and $\tau_{A}$ and $\tau_{B}$ determine the threshold proportions of variant *A* and variant *B*, respectively, that are required for the most productive coinfections. This function results in productivity increasing linearly from *W_B_* at *p_A_*=0 to *W_M_* at *p_A_ *=* *$\tau_{A}$, and then decreases linearly from *W_M_* at *p_A_* *=* $\tau_{B}$ to *W_A_* at *p_A_*=1 (Fig. 1b). Throughout the rest of the paper, we use ‘coinfection benefit’ to refer to *W_M_*, the relative productivity of mixed infections relative to the most productive single infection. Initially, we assume that the highest coinfection benefit (*W_M_*) occurs when both variants infect the host cell in equal proportion ($\tau_{A}=\tau_{B}=0.5$; Fig. 1b). However, we later relax this assumption.

We assume that, in mixed infections, the virions produced can contain the genome of either variant. Initially we assume that variants *A* and *B* are replicated and encapsidated at the same rate inside a mixed infection, and so the ratio of virions leaving the cell containing each variant’s genome is the same as the initial ratio of virions of each variant that infected the cell. However, we later relax this assumption and allow the two variants to benefit differently from the virions produced by cells in mixed infection. To do this we model the output ratio of *A*:*B* with the function $\Pi \left(P_{A}\right)={p_{A}}^{{\left(W_{A}/W_{B}\right)}^{\psi }}$, where *p_A_*, *W_A_*, and *W_B_* are as defined above, and ψ is a parameter that determines the shape of the relationship between input and output proportions of virions. When ψ is positive, it indicates that the variant which is more productive in a pure infection gains a greater share of the virions in a mixed infection; when ψ is negative it indicates that the variant which is more productive in pure infection gains a smaller share of the virions in a mixed infection (Fig. 1c). This allows us to capture a range of biological scenarios, including defective interfering particles (DIPs), which have negligible productivity in pure infection (*W*_DIP_* *=0), but gain a disproportionate share of the productivity of a mixed infection (ψ<0).

### 2.3 Dynamics

In order to determine the dynamics of variants *A* and *B*, we write an expression for the rate of change in the relative abundance of variant *A* within the host (*x*) over time:

(1) $$x_{t+1}=\frac{\sum_{k=1}^{m}\left(P\left(k\right)(\sum_{i=1}^{k}B\left(x_{t},k,i\right)(\Phi \left(\frac{i}{k}\right)\Pi (\frac{i}{k})))\right)}{\sum_{k=1}^{m}\left(P\left(k\right)(\sum_{i=0}^{k}B\left(x_{t},k,i\right)\Phi \left(\frac{i}{k}\right))\right)}$$

Here, *P(k)* gives the relative likelihood of different numbers of virions infecting each host cell (Fig. 1a), *B*(*x_t_, k, i*) gives the relative likelihood that *i* A-virions infect a host cell that is infected by *k* total virions, *Φ*(*i/k*); gives the relative productivity of a host cell infected by *i*/*k* variant *A* virions (Fig. 1b), and Π(*i/k*) gives the proportion of variant *A* virions produced by a host cell infected by *i/k* variant *A* virions (Fig. 1c).

The numerator of Equation (1) captures all of the potential ways in which variant *A* can be produced in each timestep, weighted by the relative likelihood of each of these ways. The denominator captures all of the ways in which either variant can be produced per timestep. Equation (1) therefore gives the relative frequency of variant *A* in the next generation as a function of the relative frequency of variant *A* in the current generation.

### 2.4 Equilibrium model results

**Figure 2.**
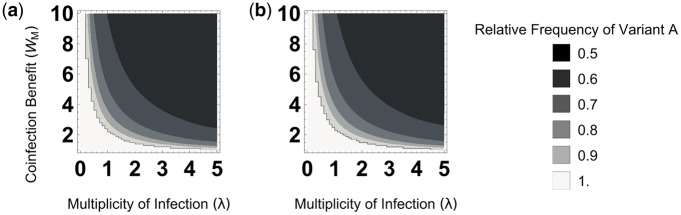
Coexistence. The *x*-axis is the multiplicity of infection (MOI; *λ*), which represents the ratio of virions to host cells. The *y*-axis is the productivity of mixed infections (*W_M_*), relative to the productivity of a cell infected only with genome A (*W_A_*). (a) Variant *A* spreads 10 times more quickly than variant *B* in pure infections (*W_A_*=1, *W_B_*=0.1). Coexistence is favoured by high multiplicity of infection (*λ*) and productivity in mixed infection (*W_M_*). (b) Variant *A* spreads 1,000 times more quickly than variant *B* in pure infections (*W_A_*=1, *W_B_*=0.001). Even though variant *A* is three orders of magnitude more productive than variant *B* in pure infection, provided coinfections are frequent and beneficial enough, variants *A* and *B* coexist at approximately equal frequencies.

**Figure 3.**
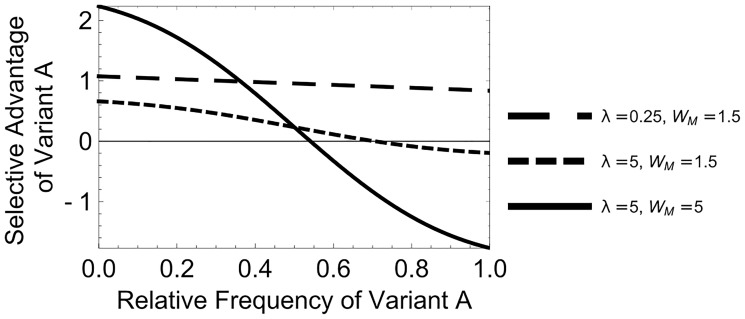
Negative frequency dependence. The selective advantage of variant *A* is plotted against the relative frequency of variant *A* in the population. As variant *A* becomes more common, its relative fitness decreases (negative frequency dependence). When the selective advantage of variant *A* is >0 it will increase in frequency, and when it is <0, it will decrease in frequency.

**Figure 4.**
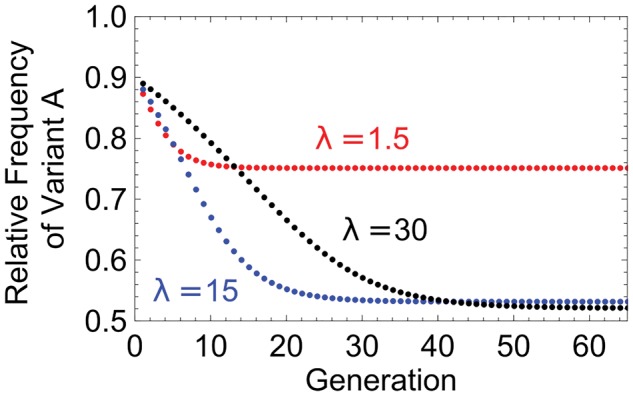
Time to reach equilibrium. The relative frequency of variant *A* is plotted against time, for different multiplicities of infection (MOI; *λ*). At low MOI (red), the system quickly reaches an equilibrium state. At higher MOI (black and blue), the system reaches an equilibrium that is closer to an even distribution of the two variants. At the highest MOI (black) it takes longer to reach this equilibrium. Therefore, while the highest MOI gives the most even equilibrium ratio of *A*:*B*, if the system is observed before it has reached equilibrium (e.g. generation 30), higher MOI may result in a more uneven ratio of *A*:*B*.

**Figure 5.**
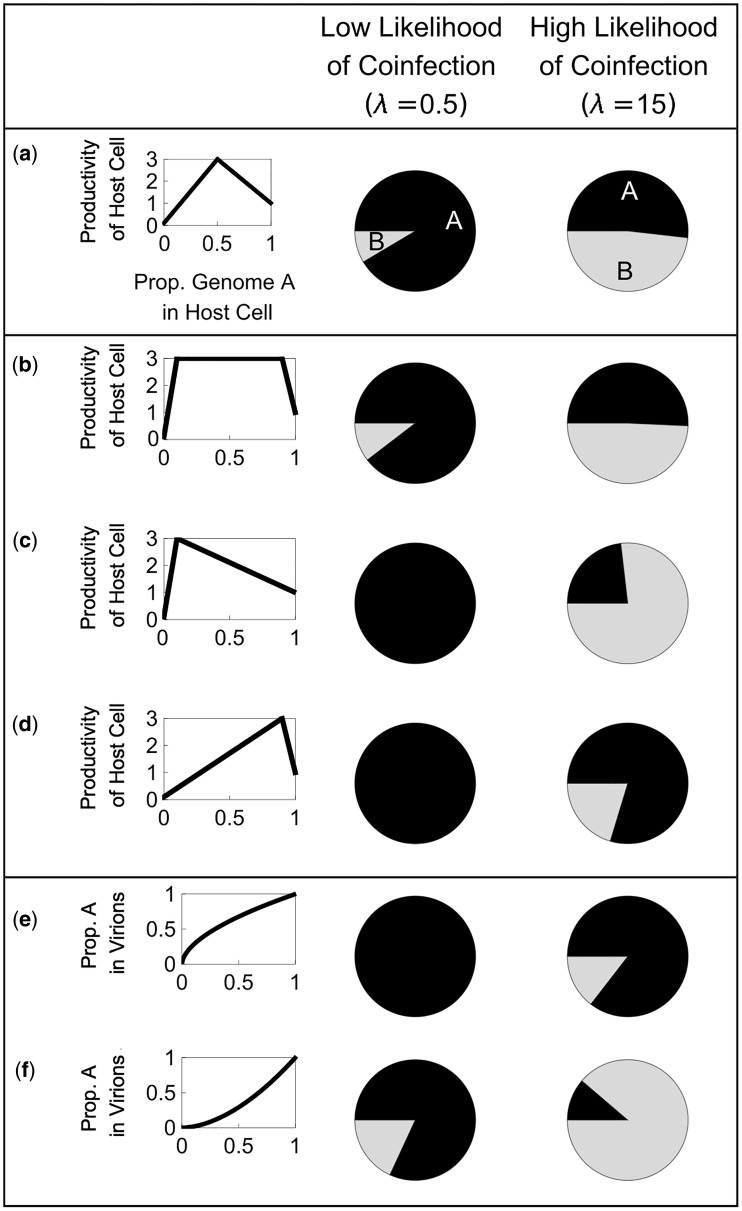
Within-cell processes. Shaded pie charts illustrate the relative frequency of each variant at equilibrium. (a) When both variants contribute equally to coinfection benefit, variant *A* dominates at low MOI, while both variants coexist at high MOI. (b) A similar pattern is seen when a small amount of either a variant maximises coinfection benefit. (c) When a small amount of the more productive variant (A) maximises coinfection benefit, variant *A* dominates at low MOI while variant *B* dominates at high MOI. (d) When a small amount of the less productive variant (B) maximises coinfection benefit, variant *A* dominates at both low and high MOI. (e) When the more productive variant (A) gains a greater share of the virions produced in mixed infection, variant *A* dominates at both low and high MOI. (f) When the less productive variant (B) gains a greater share of the virions produced in mixed infection, variant *A* dominates at low MOI but variant *B* dominates at high MOI. Overall, asymmetries in how each variant contributes to and benefits from coinfection benefit tend to disfavour coexistence.

We want to determine when variants *A* and *B* coexist stably. Therefore, we solve *x_t+_*_1_ *= x_t_* and find stable values of *x_t_* which are between 0 and 1. When a stable solution is found in this region it indicates that both genotypes are maintained within the host at an equilibrium frequency. Through a thorough search of the parameter space that we explore, we find that when an equilibrium frequency exists between 0 and 1, it will be reached from any initial frequency of the two variants. Therefore, the findings that we present here do not rely on assumptions about the initial frequencies of the two variants. Our general method was to find numerical solutions to Equation (1) for different sets of parameter values, as plotted in Figs 2–5, since finding a general analytical solution was not possible. However, our analytical solutions for a version of Equation (1) that uses a simpler function to determine coinfection are consistent with our numerical findings (Supplementary Fig. S1).

#### 2.4.1. Beneficial coinfection promotes coexistence

We find that coexistence is favoured by high coinfection benefit (*W_M_ *>* W_A_*) and high MOI (*λ*) (Fig. 2; Supplementary Fig. S2). The requirements for an appreciable MOI and coinfection benefit depended upon each other: when coinfection benefit was large, and mixed infections were an order of magnitude more productive than pure infections (*W_M_ *≫ *W_A_*), coexistence could be maintained at relatively low MOI (*λ *= 0.5–1; Fig. 2); when coinfection benefit was smaller, and mixed infections were only slightly more productive than the most productive pure infections (*W_M_*=*W_A_*), coexistence required relatively high MOI (*λ *>_** **_2; Fig. 2). Additionally, coexistence was relatively unaffected by the productivities of pure infections of variant *A* and variant *B* (*W_A_* and *W_B_*; Fig. 2). This meant that coexistence could occur even when pure infections of variant *A* were orders of magnitude more productive than pure infections of variant *B* (*W_A_ *≫* W_B_*; Fig. 2b).

Negative frequency dependence arises because when a variant is rare, it usually experiences a mixed infection together with genomes from the more common variant. In contrast, when a variant is common, it mostly experiences pure infections, with multiple genomes of its own variant. Since we assumed that mixed infections are more productive than pure infections, the rarer variant therefore experiences a higher average productivity, and subsequently has a higher mean fitness, than the more common variant (Fig. 3). This mechanism requires coinfection to be sufficiently common; when coinfection is rare and most host cells are only infected by a single virion, then the variant which has a higher productivity in pure infection has a higher average productivity and can drive the less productive variant extinct (Fig. 3). In our model, fitness refers to the expected number of progeny belonging to an individual sequence of each variant. To obtain this, we calculated the total production of each variant, and divided by the abundance of the variant; for full details see the Supplementary data.

We checked that it is this ratio of mixed to pure infections, and not the mean number of viral genomes per cell, that determines coexistence. To do this, we used a truncated Geometric function to determine the likelihood of different infection states (Godfray, O'Reilly, and Briggs 1997 ). In this function, coinfection likelihood and number of genomes per cell can be varied independently, and we were able to obtain analytical solutions using this function. We found that the maximum number of genomes per cell makes a very small difference to coexistence, whereas the likelihood of coinfection makes a very big difference (Supplementary Fig. S1). In reality, many different factors can influence the likelihood that multiple viral genomes infect each host cell, including superinfection exclusion and collective infection (Doceul et al. 2010; Folimonova 2012; Bergua et al. 2014; Díaz-Muñoz, Sanjuán, and West 2017; Sanjuán 2017, 2018; Erickson et al. 2018). Our results suggest that beneficial coinfection depends on the relative likelihood that multiple different viral genomes infect a host cell, regardless of the route by which this occurs.

### 2.5. Equilibrium model extensions

We next consider two extensions to our equilibrium model which might change the predicted level of coexistence. First, we consider bottlenecking, and then we consider asymmetries in how the two variants contribute to, and benefit from, coinfection.

#### 2.5.1 Bottlenecking disfavours coexistence

So far, we have assumed that the ratio of viral particles to host particles (MOI) remains constant throughout an infection. In reality, the MOI changes over the course of an infection, and viral populations can go through strong bottlenecks (Wilke, Reissig, and Novella 2004; Gutiérrez et al. 2010, 2015; Zwart and Elena 2015; McCrone and Lauring 2018). These changes in MOI could influence the likelihood of multiple infections, and consequently change the conditions when coexistence is favoured. Since there are many ways in which MOI could vary in reality, we do not simulate specific cases. Instead, we examine how the frequency-dependent process outlined in this paper operates at different MOIs.

We found that as MOI increases, the equilibrium frequency of *A*: *B* becomes more even, but the time it takes to reach equilibrium increases (Fig. 4). This occurs because at high MOIs, most cells are infected by at least one of each genotype. Therefore, both variants benefit from most infections, and so it takes longer for variants to change in frequency. Regular bottlenecking events could therefore have a bigger influence on the overall frequency of *A*:*B* at very high MOI than at low MOI, because the system takes longer to return to equilibrium after a perturbation. However, it is worth noting that we only saw appreciable differences in the time to equilibrium at very high MOI (*λ *> 15), and so in reality this may only matter in cases where such MOIs are typically very high, such as in tissue culture or in some plant viruses (Wilke, Reissig, and Novella 2004; Gutiérrez et al. 2010, 2015).

Bottlenecking may have additional effects that we do not consider in this analysis. For example, if a bottleneck results in a temporary reduction in viral population size, then by chance one variant could be lost from the viral population. In this case, coexistence would only be observed when the lost variant has been regained, for example, through mutation. We do not consider this stochastic effect of bottlenecking since it requires making specific assumptions about bottleneck sizes and the rates of spontaneous generation of the different variants.

#### 2.5.2 Unequal coexistence

So far, we have found that the variants tended to coexist in approximately equal proportions at high MOI and high coinfection benefit. This may reflect our assumptions that host cells produce the most virions when infected with an equal mixture of the two types, and that both variants receive a fair share of the productivity of mixed infections. In the next two sections, we relax these assumptions.

#### 2.5.3 Productivity thresholds

We examined the consequences of allowing the variants to contribute differently to coinfection benefit, by varying the ratio of *A*:*B* at which cells are most productive (*W_M_*). We did this in three different ways (Fig. 5b–d).

First, we assumed that productivity ‘plateaus’ such that only a small proportion of either genome is required for the highest coinfection benefit (Fig. 5b). We found that this leads to a slightly higher level of coexistence being maintained at both high and low MOI (Fig. 5b). This is because a higher proportion of mixed infections have the maximal productivity, so mixed infections exert a slightly stronger frequency dependent effect. When coinfection was very common (high MOI), we found that the equilibrium ratio of variant *A* to variant *B* approached 0.5.

Second, we assumed that a small amount of the more productive variant (*A*), but a large amount of the less productive variant (*B*), is required for the highest coinfection benefit (Fig. 5c). We found that when coinfection was relatively rare (low MOI), coexistence was disfavoured. This was because the most productive mixed infections occurred when lots of *B*-virions, and few *A*-virions, infected host cells. This outcome is unlikely when coinfections are rare, as variant *A* is always more frequent than variant *B*. Therefore, mixed infections were on average less productive than when the optimal threshold was more even (Fig. 5a). This lower average productivity of mixed infections leads to a lower equilibrium frequency of the variant which is weaker in pure infection (*B*).

However, we found a different result when coinfections were very common: at high MOI, the equilibrium frequency of variant *A* decreased below 0.5, favouring the variant that was less productive in pure infection (*B*). This occurred because when coinfections dominate, the overall frequency of the variants in the population depends on the virions released by mixed infections. Mixed infections which have a lower frequency of variant *A* release more virions because they are closer to the optimal A-genome frequency of 0.1, and they release more variant *B* virions than variant *A* virions. Therefore, when coinfections dominated, the overall frequency of the two genomes approached the ratio which maximises productivity in a mixed infection. In this case, this ratio resulted in more of variant *B* than variant *A*.

Third, we assumed that a small amount of the less productive variant (*B*), but a large amount of the more productive variant (*A*), is required for maximal productivity (Fig. 5d). In this scenario, coexistence was again disfavoured when coinfection was relatively rare (low MOI). This was because the most productive mixed infections were most likely to occur when *B* was rare (0.1 relative frequency). Therefore, variant *B* was unable to increase in frequency much above this value, because as variant *B* became more common, mixed infections became on average less productive, and so variant *B* was selected against.

When coinfections were very common (high MOI), coexistence was once again disfavoured: variant *A* became more common than variant *B* (Fig. 5d). This occurred for the same reason as before; when coinfection is very common, the equilibrium frequency of the two variants approaches the ratio that leads to the highest productivity of mixed infections. However, this process is not able to drive variant *B* extinct, since cells with a small fraction of variant *B* produce more virions than cells with no variant *B*.

Overall, these findings suggest that the equilibrium frequency of the two variants can be influenced by the ratio of the two variants that leads to maximum mixed productivity (*W_M_*). When this ratio is asymmetric, implying that a higher proportion of one variant is required than the other, coexistence is disfavoured at both high and low MOI. Furthermore, the variant required in the smaller proportion for maximum productivity will persist at a lower relative frequency at high MOI.

#### 2.5.4 Within-cell competition

We investigated the consequences of allowing one variant to gain a disproportionate share of the virions produced in a mixed infection. This could be the case if one variant’s genome replicates faster within a cell, for example if it is shorter, or if one variant’s genome is incorporated into virions at a faster rate. We consider two cases (Fig. 5e and f). First, we examined when the variant that is more productive in pure infection (*A*) produces a greater share of the virions in mixed infections (Fig. 5e). This could be the case if variant *A* replicates more efficiently than variant *B*, and so produces more genome copies than *B* in both pure and mixed infections. We find that in this case, the variant that does better in both pure and mixed infections (*A*) is more likely to outcompete the other variant (*B*), and so coexistence is disfavoured over the whole parameter space (Fig. 5e).

We then considered the opposite scenario, where the variant that is less productive in pure-infection (*B*) gains a greater share of the productivity of mixed infections (Fig. 5f). This could be the case if variant *B* lacks a key gene and consequently has a shorter genome, for example, if it is a DIP. In this case variant *B* could produce fewer viable virions when in pure infection, but might replicate more rapidly than genome A when in mixed infection. In this scenario, coexistence is favoured at low MOI, since mixed infections provide a stronger frequency dependent force to counteract variant *A*’s pure-infection advantage. However, when coinfection becomes common, variant *B* is able to out-compete variant *A*, reducing diversity in the opposite direction (Fig. 5f). At very high MOI, variant *B* can even drive variant *A* extinct, which agrees with previous theoretical predictions of DIP dynamics (Szathmáry 1993; Kirkwood and Bangham 1994; Frank 2000; Nee 2000; Chao and Elena 2017).

Overall, these findings suggest that when one variant benefits more from coinfection than the other, coexistence is generally disfavoured. When both variants do coexist, then the variant which gains more from mixed infection is likely to reach a higher relative frequency, even if that variant has a lower productivity in pure infection.

## 3. Parameterising the equilibrium model

We examined whether our equilibrium model led to coexistence when parameterised with real data. A caveat here is that we have not developed a model for a specific species, and there are important biological features, such as spatial structure, that we have left out of the equilibrium model. Also, we need to infer the parameters indirectly. Consequently, our aim here is to test the extent to which a specific case can be accounted for with just the simple processes included in our equilibrium model, and with data that we hope to be the right order of magnitude.

We obtained data from the literature on the H3N2 strain of human influenza A virus. In one study, two variants, that differed at a single amino acid residue in neuraminidase, D-151 and G-151, coexisted at approximately equal frequencies across multiple serial passages in tissue culture (Xue et al. 2016). We are interested in whether this pattern of coexistence can be explained through the negative frequency dependence described by our model. To do this, we need to estimate MOI and coinfection benefit.

The initial MOI is determined by the researchers and fixed at 0.2 at the start of each serial passage. As the infection progresses, the MOI will increase, since the number of viral particles increases while the number of susceptible host cells decreases. This change in MOI was not recorded, and is difficult to infer from the parameters that were recorded. However, previous theoretical work has used an MOI of 10 to reflect the higher MOI values reached over the course of a tissue culture infection (Wilke, Reissig, and Novella 2004). To allow for a conservative test of our model, we will consider MOI in the range 0.2–10 for the first viral growth phase recorded, between 8 and 16 h post-infection. If we assume higher MOI values in the initial growth period, this decreases our estimate of how productive mixed infections are. Therefore, considering an MOI as high as 10 leads to a conservative estimate for coinfection benefit, which decreases the likelihood that our model will predict coexistence.

The magnitude of coinfection benefit depends on the relative productivities (analogous to cellular *R*_0_) of host cells infected by either or both variants. Although we cannot estimate all of the parameters which contribute to cellular *R*_0_, we can infer differences in the fastest viral growth rate, *r*, observed in pure and mixed populations. We account for the fact that in the mixed population, some host cells will be infected by just one variant, by using our upper- and lower-bound estimates for MOI 8–16_** **_h post-infection (0.2–10) and the Poisson function to determine the proportion of host cells in the mixed population treatment that were infected by both variants (full details are in the Supplementary data). We therefore obtain the following estimates for the relative productivities of cells infected by only *D*, only *G*, or both *D* and *G*: *W_D_ *=1*; W_G_ *=0.007*; W_M_ *=3.2 (if MOI=10) or 59 (if MOI=0.2).

With these parameter values, both our upper- and lower-bound estimates for coinfection benefit (58.1 and 3.2, respectively) predict appreciable coexistence between D-151 and G-151 provided MOI is above 2 (Supplementary Fig. S3). Xue et al. (2016) found that coexistence was found between the variants in serial passages starting with an MOI of 0.2. Our upper-bound estimate for coinfection benefit predicts that the stable equilibrium at MOI=0.2 contains both variants whereas our lower-bound estimate predicts that variant D should out-compete variant G when the MOI is 0.2. However, if we take into account the fact that MOI is likely to increase over the course of the experiment, then both of our estimates for coinfection benefit predict stable coexistence between the two variants, provided that the MOI increases above 0.7 before variant G is lost from the population. Consequently, our model shows that, even with relatively rough calculations of the relevant parameters, to parameterise a relatively simple model, beneficial coinfection can explain the coexistence of multiple genetic variants. The data suggest that D-151 and G-151 coexist at roughly equal frequencies across multiple passages (Xue et al. 2016). Our model predicts that D will be slightly more common, even at high MOI, but there are many parameters, that we have not been able to estimate, which will influence the ratio of the two variants, and our model was not designed to match this specific system (Fig. 5). Our aim here was test our model qualitatively (can it explain coexistence?), not quantitatively (what fraction will be variant D-151?).

## 4. The spatial simulation 

So far, we have taken a relatively simple approach that has allowed us to investigate the role of coinfection likelihood and coinfection benefit in the maintenance of viral diversity. However, this approach is limited in two key ways. Firstly, we have assumed that the relative frequency of each variant and the ratio of virions to susceptible host cells (MOI) are independent. However, if coinfection benefit is above one, then the viral population will increase in size as the ratio of the two variants becomes more even. Consequently, if the MOI in the equilibrium model is taken as the starting MOI, then our model is likely to underestimate the degree of coexistence arising from different levels of coinfection benefit. Secondly, our model does not include spatial structure, which could play an important role in influencing the likelihood of coinfections involving both variants. On the one hand, spatial structure could increase coexistence, since the different viral variants would infect different cells, and so they might not be in direct competition. On the other hand, spatial structure could decrease coexistence, by reducing the likelihood that host cells are infected by both variants, and so reducing the importance of coinfection synergy. To test whether our key predictions still hold when these factors are taken into account, we applied our model in a spatial simulation of viral growth in a two-dimensional grid of cells.

### 4.1. Simulation description

**Figure 6.**
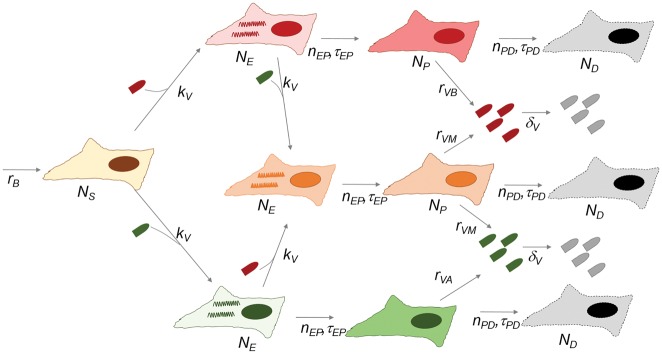
Scheme of the simulation. A two-dimensional grid contained *N_S_* susceptible, *N_E_* eclipse phase, *N_P_* virus-producing, and *N_D_* dead cells. Infection (*N_S_* → *N_E_*) was a second order, Poisson stochastic processes occurring with probability *P = *1 − exp(−λ*Δt*) for each cell and simulation time unit *Δt* (0.1 min). For infection, λ*=k_V_VN_S_*, where *k_V_* is the infection rate (infectivity), *V* is the local virus concentration, and *N_S_* is 1 or 0.

We have a diffusion-reaction model which we parameterised using values typical for a fast-replicating lytic animal virus. We considered a population of cells in a two-dimensional grid, each of which could be susceptible, infected but not yet producing viruses (eclipse phase), producing viruses, or dead (Fig. 6). We can consider one, two or several virus variants, with characteristics that we can control. Cells can be infected by one or several variants of the virus, and we can use different models to calculate the number of virions of each variant produced by multiply infected cells. We modelled infection as a second-order, Poisson stochastic process that depended on binding to virions. All other cellular state transitions were first-order random processes occurring at a fixed mean time. Infection spread was governed by a diffusion-reaction process in the two-dimensional grid of cells.

In order to generate a chronic infection model, we established values of cell supply rate (*r_B_*) and virus outflow rate (*δ_V_*) that resulted in a stable equilibrium concentration of viruses and cells. In order to calculate a stable MOI, we adapted the infectivity of the virions (*k_V_*). The generation time is the mean eclipse time plus the mean virus production time (*τ_EP_+τ*_PD_; here 12 h). The infection probability is: *P***_* *_***=***_* *_**1 − exp(−MOI) = 1 − exp(−λΔ*t*). So we have that MOI = λΔ*t* and we take Δ*t=*12 h. In the model, λ*=k_V_VN_S_*, where *N_s_* can only be 0 or 1 (which means that, in a given grid subunit, a cell is either susceptible or unsusceptible/dead) and *V* is the local virion concentration. This way, we control the MOI using the infectivity parameter, *k_V_*, but, unlike in the equilibrium model, the MOI is also affected by the virus equilibrium concentration (*V*). The dynamics of *V* are described by a reaction–diffusion process of the form ∂*V/*∂*t=r*_v_*N_p_*−δ_V_*V* *+* *D*Δ*V*, where *N_P_* are producer cells (0 or 1, as above), *r_v_* is the virus production rate of infected cells, *δ_V_* is the virus degradation/outflow rate, *D* is the diffusion coefficient, as defined by the Stokes–Einstein equation, and Δ*V* is the virus concentration gradient (we ignored loss of viruses due to adsorption). We perform dynamic simulations to find the equilibrium coexistence between variants with different fitness values, so we can investigate coexistence when both infectivity and viral population growth can influence MOI. We allow variant *A* and variant *B* to have different fitness values by scaling *r_V_*, the rate at which virions are produced in cells, by *W_A_* or *W_B_*. We allow for coinfection benefit by allowing cells infected by both variants to have the highest *r_V_* values. Parameter values used are shown in Supplementary Table S1 and correspond to a typical fast-replicating lytic animal virus.

### 4.2. Simulation results

**Figure 7.**
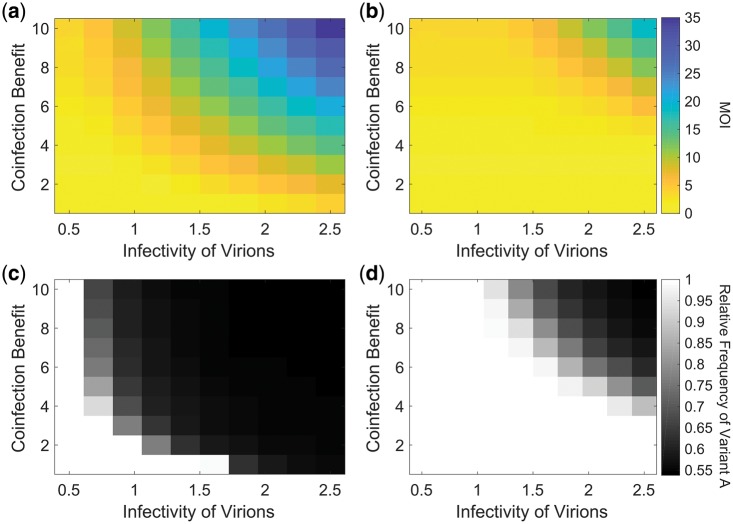
Coexistence in the simulations. The heatmaps represent equilibrium values, which were typically reached after 20 generations. In (b) and (d), superinfection exclusion occurs 3 h after a cell is infected. (a) The final MOI (ratio of viral particles: susceptible host cells) depends on both the infectivity of viral particles and the coinfection benefit. The infectivity is the likelihood that a viral particle will successfully infect a host cell upon contact. (b) Superinfection exclusion reduces the MOI since it makes multiple infection less likely. (c) Coexistence between the two variants is most likely when the coinfection benefit is large and viral particles are highly infectious. (d) Superinfection exclusion reduces the parameter space under which coexistence is found. All parameters were as shown in Supplementary Table S1 except those varied in the graph; *r_v_* was 10 times lower for variant *B*.

We found that, as predicted, the MOI increased as the infection progressed, and that the final MOI reached depended on both the coinfection benefit and the infectivity of viral particles (Fig. 7a). The degree of coexistence between the two variants was therefore determined by both the coinfection benefit and infectivity (Fig. 7c). We also investigated superinfection exclusion, using a superinfection exclusion time of 3 h post-infection. We found that superinfection exclusion resulted in a lower MOI, which reduced the parameter space under which coexistence occurred, although we still found coexistence at high coinfection benefit and when viral particles were highly infectious (Fig. 7b and d). Our simulation generally reached an equilibrium quickly, within twenty viral generations, which indicates that at least in a two-dimensional infection process, spatial structure may only temporarily reduce the likelihood of multiple infection. We further confirmed that both MOI and coinfection benefit contributed to coexistence in the simulation (Supplementary Fig. S4).

Overall, the simulation results agree with the predictions from the equilibrium model that coinfection benefit and the likelihood of multiple infection can contribute to coexistence and highlight that different factors can contribute to the likelihood of multiple infection.

## 5. Discussion

We investigated theoretically how mutually beneficial interactions between viral variants influence coexistence. We found that coexistence could occur when mixed infections were frequent relative to pure infections and when they were more productive than pure infections (Figs 2, 3, and 7; Supplementary Figs S1 and S2). This effect did not depend on the initial frequencies of the two variants and it was able to counteract even very significant fitness differences between variants when in pure infections (Fig. 2b). Furthermore, we found that when coinfections were very common, coexistence between variants was determined by two factors: the ratio of the two variants that maximised productivity in mixed infections (Fig. 5b–d); and the relative benefit each variant gains in mixed infections (Fig. 5e and f). We parameterised our model using data from the H3N2 strain of human influenza A virus, and found that it could explain coexistence (Supplementary Fig. S3) (Xue et al. 2016). We also developed a more realistic spatial simulation, and found that in this, mutually beneficial interactions also led to coexistence (Fig. 7).

The extent to which our model predicts coexistence depends upon two main factors. First, coexistence requires coinfection of the same cell by the different variants. Empirical estimates have found that MOI, and hence the possibility for coinfection, is higher in plant viruses and in tissue culture, which could explain why empirical examples of beneficial coinfection mostly come from observational studies on plant viruses, or from cell culture experiments on animal viruses (Wilke, Reissig, and Novella 2004; Gutiérrez et al. 2010; Shirogane, Watanabe, and Yanagi 2012; Bordería et al. 2015; Xue et al. 2016, 2018). Second, we require that mixed coinfections are more productive than pure infections. Both large and small coinfection benefits have been observed, and these frequently arise when virions from cells in mixed infection are more effective because they contain proteins encoded by multiple viral genomes (‘phenotype mixing’) (Závada 1976; Roossinck, Sleat, and Palukaitis 1992; López-Ferber et al. 2003; Hull 2009; Shirogane, Watanabe, and Yanagi 2012; Border**í**a et al. 2015; Xue et al. 2016). One common cause of coinfection benefit could be if beneficial mutations exhibit antagonistic epistasis when in the same genome, but not in different genomes—this appears to occur more commonly in RNA viruses than other organisms (Holmes 2003; Sanjuán and Elena 2006).

Our model highlights how the evolutionary consequences of coinfection depend on the details of how gene products are shared in coinfection. In our model, gene product mixing is synergistic, allowing for beneficial coinfection in which cells infected by both variants are more productive than cells infected by either variant alone. Previous models have either assumed full complementation, in which cells infected by both variants have the same productivity as cells infected by just one variant, or intermediate complementation, in which cells infected by both variants have the mean productivity of cells infected by either variant alone (Chao 1991; Szathmáry 1993; Kirkwood and Bangham 1994; Godfray, O'Reilly, and Briggs 1997; Frank 2000; Bull, Godfray, and O'Reilly 2001; Wilke and Novella 2003; Novella, Reissig, and Wilke 2004; Wilke, Reissig, and Novella 2004; Gao and Feldman 2009). Consequently, these previous models require another mechanism to explain stable coexistence, such as the less fit variant being able to exploit the other (Szathmáry 1992; Kirkwood and Bangham 1994). We have shown that synergistic mixing of gene products, which we have called beneficial coinfection, is enough on its own to allow for the stable coexistence of different variants (Fig. 2).

We found that when coinfection was common, variants that gained a greater share of coinfection could reach very high frequencies, even if they had very low productivity in pure infections. This potential advantage was greatest when coinfection benefit was highest, and so beneficial coinfection could favour mutants that trade off productivity in pure infection for a greater share of the virions produced in coinfection. This could lead to greater selection for DIPs, which lack key genome sections, and so are unable to replicate in pure infections, but can gain a disproportionate advantage in mixed infections (von Magnus 1954; Huang and Baltimore 1970; Nee and Maynard Smith 1990; Szathmáry 1992, 1993; Pathak and Nagy 2009; Nee 2016). Therefore, it is possible that beneficial coinfection could promote coexistence only transiently, with mutually beneficial variants eventually being replaced by DIPs, or that cycles would occur with wild-types evolving resistance to DIPs (DePolo, Giachetti, and Holland, 1987).

Our model can also be applied to help explain the evolutionary stability of multipartite viruses. Multipartite viruses have a genome which is split into multiple segments, and each segment is packaged into a separate virion (van Kammen 1972; Fulton 1980; Lucía-Sanz and Manrubia 2017). Empirical studies have found that these different genome segments of multipartite viruses can exist at different equilibrium frequencies within a host, despite the fact that every segment is required for successful infection of host cells (Sicard et al. 2013; Hu et al. 2016; Wu et al. 2017). In our model, multipartite viruses are captured by the case where neither variant can replicate on its own (*W_A_ *=*W_B_* ∼ 0; *W_M_*>0), and so they represent an extreme example of beneficial coinfection. The potential advantages of being multipartite are captured by our coinfection benefit (*W_M_*) parameter (Iranzo and Manrubia 2012). Our model suggests two mechanisms by which multipartite virus segments could coexist at unequal frequencies within a host (Chao 1991; Szathmáry 1992; Iranzo and Manrubia 2012). The first mechanism was also suggested by Szathmáry (1992) and depends on one segment obtaining a greater benefit from mixed infection than the other (Fig. 5e and f) (Szathmáry 1992). There is evidence that different segments of multipartite viruses could achieve this through different rates of replication or encapsidation (Herzog and Hirth 1978; Loesch-Fries and Hall 1980; Dore, Pinck, and Pinck 1989). The second mechanism is that the segments contribute asymmetrically to the productivity of infected cells, such that cells are most productive when infected by an uneven ratio of segments. This could occur if segments encode different gene products which are required in different amounts. In this case, cells infected by the optimal ratio of segments will produce the most virions, and will also produce virions in this optimal ratio, provided that one genome is not encapsidated substantially faster than the other. Therefore, the equilibrium frequency of segments in the system as a whole converges upon the frequency which maximises the productivity of infected cells (Fig. 5b–d).

To conclude, there are a number of ways that our equilibrium model could be expanded, to match the biology of specific virus-host systems. One possibility is that if mutation rates are high, then natural selection can act on ‘clouds’ of mutationally linked genotypes (quasispecies theory), rather than on individual genotypes (Wilke, Reissig, and Novella 2001; Wilke 2005; Lauring and Andino 2010; Domingo, Sheldon, and Perales 2012). Our model shows how coexistence can emerge without invoking high mutation rates, and so it may be applicable in a wide range of viruses. Different modes of viral spread can also influence the likelihood of multiple infections, and so a natural extension of our model could incorporate cell–cell spread, virion aggregation, and other modes of collective infection (Sanjuán 2017, 2018). Finally, our model has focused on evolution within hosts, whereas coexistence of viral variants at the epidemiological level is likely to depend on both evolution within hosts and transmission between hosts.

## Supplementary data

Supplementary data are available at Virus Evolution online. Supplementary code is available at https://osf.io/akrmp/.

## Supplementary Material

Supplementary Data
