# Supplementary material for: Beneficial coinfection can promote within-host viral diversity
Source: Virus Evol. 2018 Oct 1;4(2):vey028. doi: 10.1093/ve/vey028 (PMC6166523; doi:10.1093/ve/vey028)
Supplement: Supplementary Data [file vey028_supp.zip › coinfection_SI_word.docx]

General Model

Model Setup

1. Coinfection

We use a Poisson distribution to determine the likelihood of a cell being infected by multiple viral particles. The Poisson parameter, *λ*, is given by the ratio of infectious viral particles to susceptible host cells (multiplicity of infection; MOI). We're only going to sum from 1 and above, because cases where a cell is infected by zero viral particles don't affect the relative frequency of the two variants, A and B.

With a Poisson distribution, potentially an infinite number of viral particles could infect each cell, with a greatly diminishing likelihood of very high numbers of viral particles. For simplicity, we decide a maximum *k* (number of genomes/cell) at which we stop summing the likelihoods given by this function. We want to avoid summing lots of probabilities which are very small when *λ* is small, but we also want to avoid missing values of k which are quite likely when *λ* is high. We therefore want to scale the max value which we sum over by *λ*. Because the variance (σ^2) of a Poisson is *λ*, we know that values of *k* which are >3*σ* higher than the mean are very unlikely (<<1%).

Therefore, we sum between 1 and $\lambda+3 \sqrt{\lambda}$, rounded up. This approximation captures the vast majority (>>99%) of potential infection states. For small values of λ, k=0 can be quite a large part of the distribution, however we're excluding it. We're interested in the relative likelihood of each infection state k, so we must divide our function P(λ) by the sum of probabilities over the relevant range (i.e. from 1 to λ+3σ). Overall, this yields $P\left( k \right)=(e^{-\lambda} \frac{\lambda^{k}}{k!}) /(\sum_{n=1}^{m} e^{-\lambda} \frac{\lambda^{n}}{n!})$, where *λ* is the ratio of virions to host cells, *k* is the number of virions infecting each host cell, and *m* is $\lambda+3\sqrt{\lambda}$; for an illustration of this function for different values of *λ*, see Fig. 1a.

We next assume that the likelihood of a cell being infected by either variant A or variant B depends on the relative abundance of each variant and is independent of other infection events. Therefore, we can describe the number of virions of variant A that infect a given cell with a Binomial function, as follows: $B\left( x_{t},k,i \right)=\binom{k}{i}{x_{t}}^{i} {{(1-x}_{t})}^{k-i}$, where $x_{t}$ is the relative frequency of variant A at time t (and so ${1-x}_{t}$ is the relative frequency of variant B), *k* is the number of virions infecting the host cell, and *i* is the number of virions infecting the host cell which contain genome A.

1. Cell Productivity

We want to investigate a scenario where two variants, A and B, produce infected host cells which differ in their productivity. Productivity refers to the relative expected number of further host cells infected by virions produced by a focal infected host cell, and so is analogous to R_0_ at the cellular level. Viral traits which result in more virions being produced, or more effective virions, both contribute to increased productivity. We also want to capture the possibility that mixed infections containing both variants are more productive than infections containing just one variant. To achieve this, we used a discontinuous distribution where we can independently specify the productivity of pure-A and pure-B infections, and the maximum productivity of mixed infections, given by:

$$\phi\left( P_{A} \right)=\left\{ \begin{aligned} {W_{B}+p}_{A}\frac{W_{M}-W_{B}}{\tau_{A}}, &0\leq p_{A}<\tau_{A} \\ W_{M}, &{\tau_{A}\leq p}_{A}\leq\tau_{B} \\ {\frac{\tau_{B}W_{A}-W_{M}}{\tau_{B}-1}+p}_{A}\frac{W_{M}-W_{A}}{\tau_{B}-1}, {\tau_{B}< p}_{A}\leq1 \end{aligned} \right.$$

Where *p_A_* is the relative proportion of genome A infecting a given host cell, W_A_ is the productivity of a cell infected only by variant A (when *p_A_* = 1), W_B_ is the productivity of a cell infected only by variant B (when *p_A_* = 0), W_M_ is the maximum possible productivity of a cell (given by some value in between *pA* = 0 and *p_A_* = 1, as determined by *τ_A_* and *τ_B_*), and *τ_A_* and *τ_B_* give the threshold proportions of genome A and genome B respectively required for the most productive cellular infections.

The maximum productivity can occur at different thresholds of each variant by varying *τ_A_* and *τ_B_*. By using $\phi\left( P_{A} \right)$ we can define the productivity of host cells infected by any combination of the two variants. This function can also allow mixed infections to be less productive than pure infections, or for mixed infections to be as productive as the 'average' productivities of the variants infecting it, as has been previously assumed by similar models (Novella et al. 2004). We plot $\phi\left( p_{A} \right)$ in Fig. 1b.

1. The Share of Productivity

Host cells infected by both variants are more productive than host cells infected by just one variant, however the benefits of this increased productivity might not be shared equally. For example, one variant may be more likely to be incorporated into virions in a mixed infection than another. To capture this, we use a function which maps the frequency of each variant infecting a cell to that variant's frequency in the virions produced by that cell: $\Pi\left( P_{A} \right)={p_{A}}^{{(\frac{W_{A}}{W_{B}})}^{\psi}}$, where *p_A_*, *W_A_* and *W_B_* are as defined above, and $\psi$ is a parameter that determines the shape of the relationship between input and output proportions of virions. This function allows for positive, negative, or no correlation between productivity in pure infection and share of benefit in mixed infection; the function is plotted in Fig. 1c.

Model Analysis

We combine the functions described above into a single expression, which relates the relative abundance of variant A in the next generation, denoted *x_t+1_*, to the relative abundance of variant A in the current generation, denoted *x_t_*:

$$x_{t+1}=\frac{\sum_{k=1}^{m} (P\left( k \right)(\sum_{i=1}^{k} B\left( x_{t},k,i \right)(\Phi\left( \frac{i}{k} \right)\Pi(\frac{i}{k}))))}{\sum_{k=1}^{m} (P\left( k \right)(\sum_{i=0}^{k} B\left( x_{t},k,i \right)\Phi\left( \frac{i}{k} \right)))}$$

The numerator of our expression for *x_t+1_* gives all of the ways variant A can be produced each generation. To calculate this, we sum over every possible infection state (i.e. whether a cell is infected by 1, 2, 3 ... viral genomes), and weight each infection state by its relative probability as given by *P(k)*. For each infection state, we sum over all of the ways variant A can be present (i.e. summing from i=1 to i=k) and we weight these by their probabilities as given by our Binomial function B(xt,k,i). For each of these infection states i, we calculate the productivity of that host cell as given by $\phi\left( \frac{i}{k} \right)$ and the proportion of that productivity which goes to variant A, as given by $\Pi\left( \frac{i}{k} \right)$. Note that we have used the fraction of infecting genomes which are variant A, given by i/k, to determine p_A_, the proportion of infecting genomes which are variant A.

Analogously, the denominator of our expression for *x_t+1_* gives all of the ways that both variants can be produced each generation. By dividing by this, we ensure that the expression gives the change in relative variant frequency. This means that the absolute values W_A_, W_B_, and W_M_ are unimportant; what matters is their magnitude relative to one another.

Obtaining Model Results

We are interested in solving for a value of *x*, *x^*^*, for which *x_t+1_* = *x_t_*. We are unable to obtain a general analytical solution with this form of the model, so we instead solve $x_{t+1}-x_{t}=0$ for x numerically for a range of values. Over the parameter ranges that we solve for, we find that there is only ever one real solution between 0 and 1 and that it is always a maximum of the system. By solving for different parameter values as specified in figure legends, this method produces Figs. 2, 5, S2, and S3.

In Fig. 3, we plot the relative fitness of each variant. To obtain this we divide all of the production of each variant (for variant A, this is equivalent to the numerator of the expression for $x_{t+1}$), by the relative abundance of that variant. This gives a per-capita growth rate of each variant. We then express the per-capita growth rate of variant A relative to the per-capita growth rate of variant B to obtain the relative fitness of variant A:

$$W_{A}=\frac{\sum_{k=1}^{m} (P\left( k \right)(\sum_{i=1}^{k} B\left( x_{t},k,i \right)(\Phi\left( \frac{i}{k} \right)\Pi(\frac{i}{k}))))}{x_{t}}-\frac{\sum_{k=1}^{m} (P\left( k \right)(\sum_{i=0}^{k-1} B\left( x_{t},k,i \right)(\Phi\left( \frac{i}{k} \right)(1-\Pi\left( \frac{i}{k} \right)))))}{{1-x}_{t}}$$

In Fig. 4, we plot how *x* changes over time. To do this we set an initial value of x and then iterate by substituting this into the expression for $x_{t+1}$ for a set number of iterations.

Code to obtain these results is available as a Mathematica document at <https://osf.io/akrmp/>.

General Model Extension

In Fig. S1, we use a Geometric function to determine the likelihood that cells are infected by different numbers of virions. We assume that infection of host cells occurs according to a Geometric process, as in Godfray et al 1997. The number of trials (*m*) is the maximum number of viral genomes which can potentially infect each host cell. The likelihood of success, *α*, is the chance that after infection by a virion, superinfection exclusion occurs such that it's impossible for future viral genomes to infect the host cell. To model this, we use a Geometric function given by $\frac{{\alpha(1-\alpha)}^{k-1}}{1-{(1-\alpha)}^{m}}$ where *k* is the number of viral genomes infecting a cell, and *α* and m are as defined above. As before, the denominator normalises so that we’re dealing with relative likelihoods of different infection states.

To obtain results with the Geometric function, we solve for an analytical expression for *x** in terms of W_M_ and *α* for a given value of m. For example, for *m* = 3, $x^{*}=\frac{-0.05+0.5W_{M}+\frac{0.675}{2.5+\alpha(\alpha-3.5)}}{W_{M} - 0.55}$.

Spatial Simulation

We developed a diffusion-reaction model that we parameterised using typical values for a fast-replicating lytic animal virus. We used a grid of cells, in which every subunit simulates the area occupied by a single cell. The viruses were simulated as a substance that diffuses through the grid. As shown in Figure 6 of the main text, the cells can be in four states: susceptible (N_s_, not infected and susceptible to infection), eclipse (N_e_, infected but not yet virus producer), virus producer (N_p_) and dead (N_D_). During the eclipse phase, the cell can be susceptible to superinfection or not. The infection process (N_s_ 🡪 N_e_) was modelled as a Poisson process occurring with probability:

$P=1-exp(-\lambda\Delta t)$,

where Δt is the time step of the simulation and $\lambda=k_{v}V N_{s}$, where V is the viral concentration within the grid subunit, N_s_ is the number of susceptible cells (0 or 1) and k_v_ is the infectivity of the virions. When we have both viruses in the same cell, the probability was calculated as (considering P’_A_ and P’_B_ as the probabilities of the two independent infections):

$${P'}_{A}=1-exp(-\lambda_{A}\Delta t)$$

$${P'}_{B}=1-exp(-\lambda_{B}\Delta t)$$

$$P_{AB}={P'}_{A}+{P'}_{B}-{P'}_{A}\times{P'}_{B}=1-exp(-{(\lambda}_{A}+\lambda_{B})\Delta t)$$

$$P_{A}=P_{AB}\times\frac{{P'}_{A}}{{P'}_{A}+{P'}_{B}}$$

$$P_{B}=P_{AB}\times\frac{{P'}_{B}}{{P'}_{A}+{P'}_{B}}$$

All other cellular state transitions were modeled as random processes occurring with cumulative distribution function:

$$P= \frac{t^{n}}{t^{n}+\tau^{n}}$$

where *τ* is the process half time and *n* the Hill coefficient controlling the steepness of the transition. Besides the transitions shown in Figure 6, we also considered the superinfection susceptibility status, i.e. during the eclipse phase cells may be susceptible to superinfection (we used the same above function to model this transition).

The dynamics of the viral concentration in every grid subunit (V(x,y)) obeyed the following partial differential equation:

$$\frac{\partial V\left( x,y \right)}{\partial t}=r_{v}N_{p}-\delta_{V}V\left( x,y \right)+D \Delta V,$$

where N_p_ are producer cells (0 or 1, as above), r_v_ is the virus production rate of infected cells, δ_V_ is the virus degradation/outflow rate, D is the diffusion coefficient as defined by the Stokes-Einstein equation, and ΔV is the virus concentration gradient (we ignored loss of viruses due to adsorption).

In the simulations, the time step was selected so that the probability of a transitions was small. The stochastic transitions were simulated with a Monte-Carlo method, and for the deterministic parts of the simulation (diffusion and reaction) we used the Euler integration method. For diffusion around the limits of the grid we used a continuous-system approximation. The parameter values of the simulation are shown in Table S1 and MATLAB code implementing this simulation is available at <https://osf.io/akrmp/>.
