# Supplementary material for: Beneficial coinfection can promote within-host viral diversity
Source: Virus Evol. 2018 Oct 1;4(2):vey028. doi: 10.1093/ve/vey028 (PMC6166523; doi:10.1093/ve/vey028)
Supplement: Supplementary Data [file vey028_supp.zip › Supp.docx]

Figure S1

Coexistence of variants with similar productivities. The x-axis is the multiplicity of infection (MOI; *λ*), which represents the ratio of virions to host cells. The y-axis is the productivity of mixed infections (*W_M_*), relative to the productivity of a cell infected only with variant A (*W_A_*). (a) Variant B spreads 0.9 times as quickly as variant A in pure infections (*W_A_* = 1, *W_B_* = 0.9). Coexistence is favoured in a similar region of parameter space as when one variant is much fitter in single infection (Fig. 2), however now, both variants exist at equal frequencies in most of the parameter space in which coexistence is favoured.

Figure S2

The coexistence of two variants when a Geometric function gives the likelihood of coinfection. Here, we assume that up to three virions can infect each host cell; the likelihood of success of each successively infecting virion is given by the likelihood of superinfection exclusion (*α*). Similar qualitative results are found to our main results (Fig. 2); a higher likelihood of coinfection favours coexistence.

Figure S3

The predicted coexistence of two variants, D-151 and G-151, as MOI varies, where coinfection benefit is inferred from experimental data. Both estimates predict coexistence provided MOI > 0.7. As MOI increases further, both estimates predict increasingly equal levels of coexistence.

Figure S4

Coexistence and MOI in the simulation. We altered the equilibrium MOI in three ways: by increasing the infectivity of virions (*k_V_*; between 0.8 and 4.64); by increasing the baseline productivity of infected cells (*r_v_*; between 4.2 and 23.1); and by increasing the coinfection benefit (*W_M_*; between 1 and 7.5). All three routes increase the MOI and consequently lead to greater levels of coexistence. However, increasing the coinfection benefit leads to a disproportionate increase in the level of coexistence, highlighting that coinfection benefit per se influences the level of coexistence. Each point plots the mean equilibrium frequency of variant A over 24 replicates +/- the standard deviation.

Table S1

Parameters used in the simulation.

^1^The size of a prototypical virus, such as vesicular stomatitis virus

^2^This parameter was varied to control viral population density and the MOI, as shown in Fig. 7. The value *k_V_* = 0.5 produced the lowest MOI value explored (ca. 0.2 viral particles/cell).

^3^Typical values for a prototypical animal lytic virus

^4^Each cell hence produces *r_V_* × *τ_PD_* = 1584 infectious particles on average, a realistic value for an eukaryotic virus.

^5^Adjusted to allow a nonzero viral density at equilibrium. The outflow rate corresponds to the degradation rate of a relatively stable virion and does not include other processes such as dilution or inactivation by the immune system. Consequently, the cell supply rate required to maintain the population is high.

| **Process** | **Parameter** | **Value** | **Units** | **Meaning** |
| --- | --- | --- | --- | --- |
| Diffusion | C | 4 × 10^–4^ | µm^–2^ | Cell density |
|  | A | 6 × 10^6^ | µm^2^ | Area of cell population |
|  | kT | 4.28 × 10^–21^ | J | Boltzmann constant × temperature |
|  | µ_w_ | 6.91 × 10^–4^ | Pa × s | Dynamic viscosity of the medium (water) |
|  | D_V_ | 0.18 | µm | Hydrodynamic diameter of the virion^1^ |
| Reaction (virus) | k_V_ | 0.5 | ([V] × min)^–1^ | Infectivity^2^ |
|  | τ_EP_ | 240 | min | Mean duration of eclipse phase^3^ |
|  | τ_PD_ | 480 | min | Mean duration of virus production phase^3^ |
|  | r_V_ | 3.3 | V × min^–1^ | Rate of virion production in infected cells^4^ |
|  | r_B_ | 3.3 x 10^-3^ | min^-1^ | Cell supply rate^5^ |
|  | δ_V_ | 5 × 10^–3^ | min^–1^ | Virus outflow rate^5^ |
