# Supplementary figures and images for: Beneficial coinfection can promote within-host viral diversity
Source: Virus Evol. 2018 Oct 1;4(2):vey028. doi: 10.1093/ve/vey028 (PMC6166523; doi:10.1093/ve/vey028)

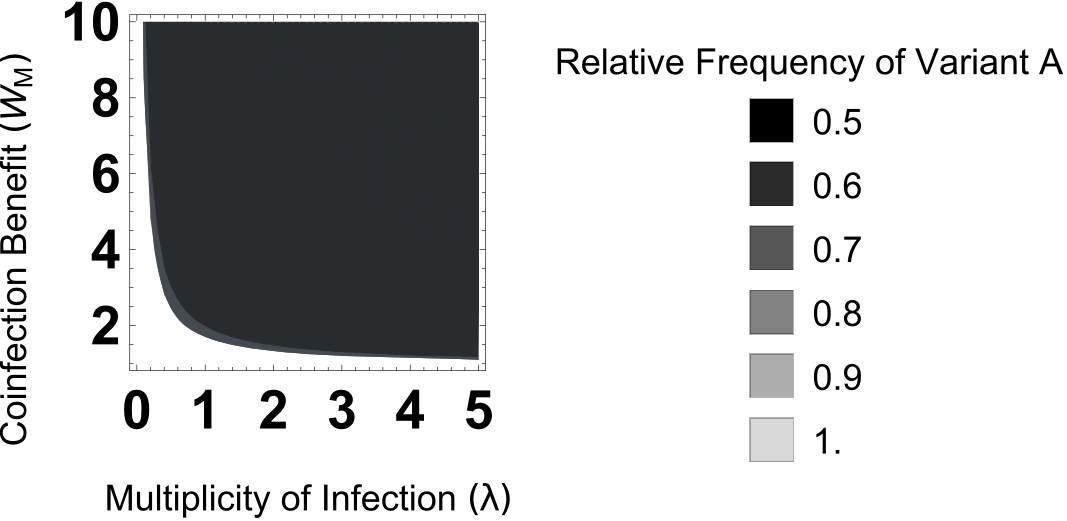

Supplement: Supplementary Data [file vey028_supp.zip › coinf_figs1.tiff]

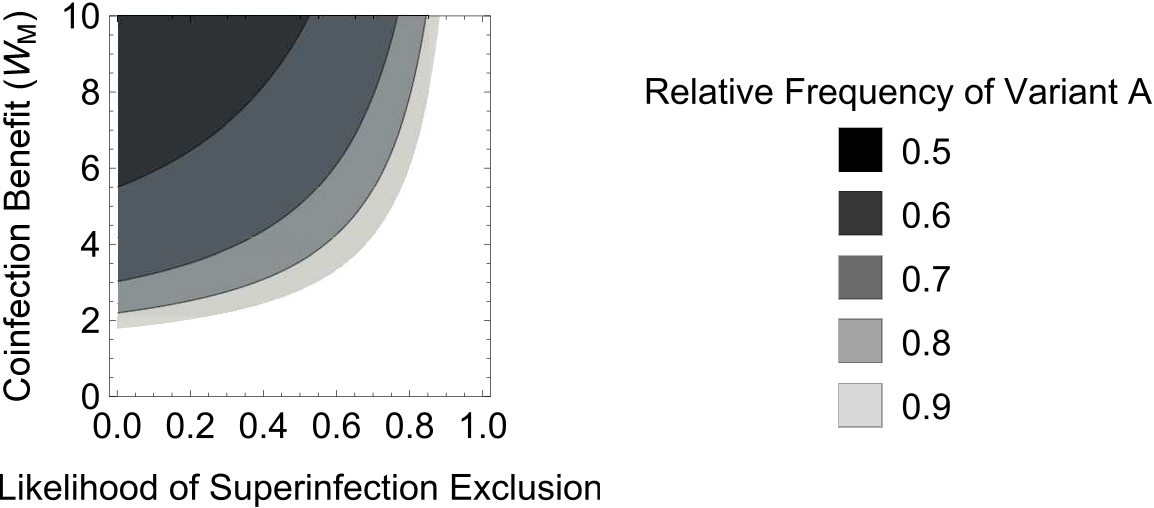

Supplement: Supplementary Data [file vey028_supp.zip › coinf_figs2.tiff]

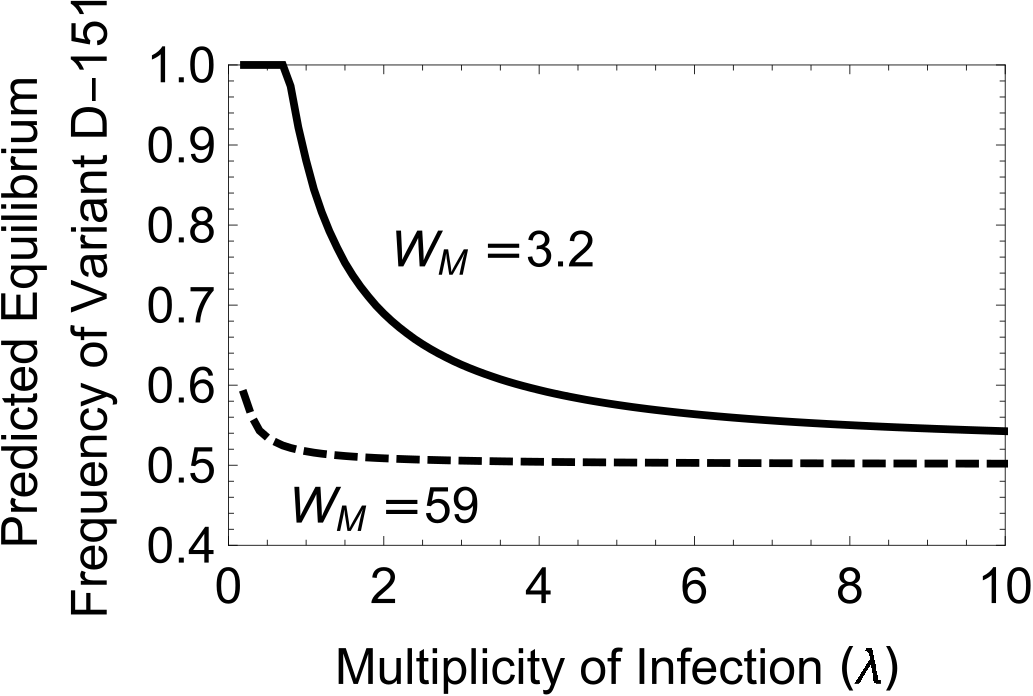

Supplement: Supplementary Data [file vey028_supp.zip › coinf_figs3.tiff]

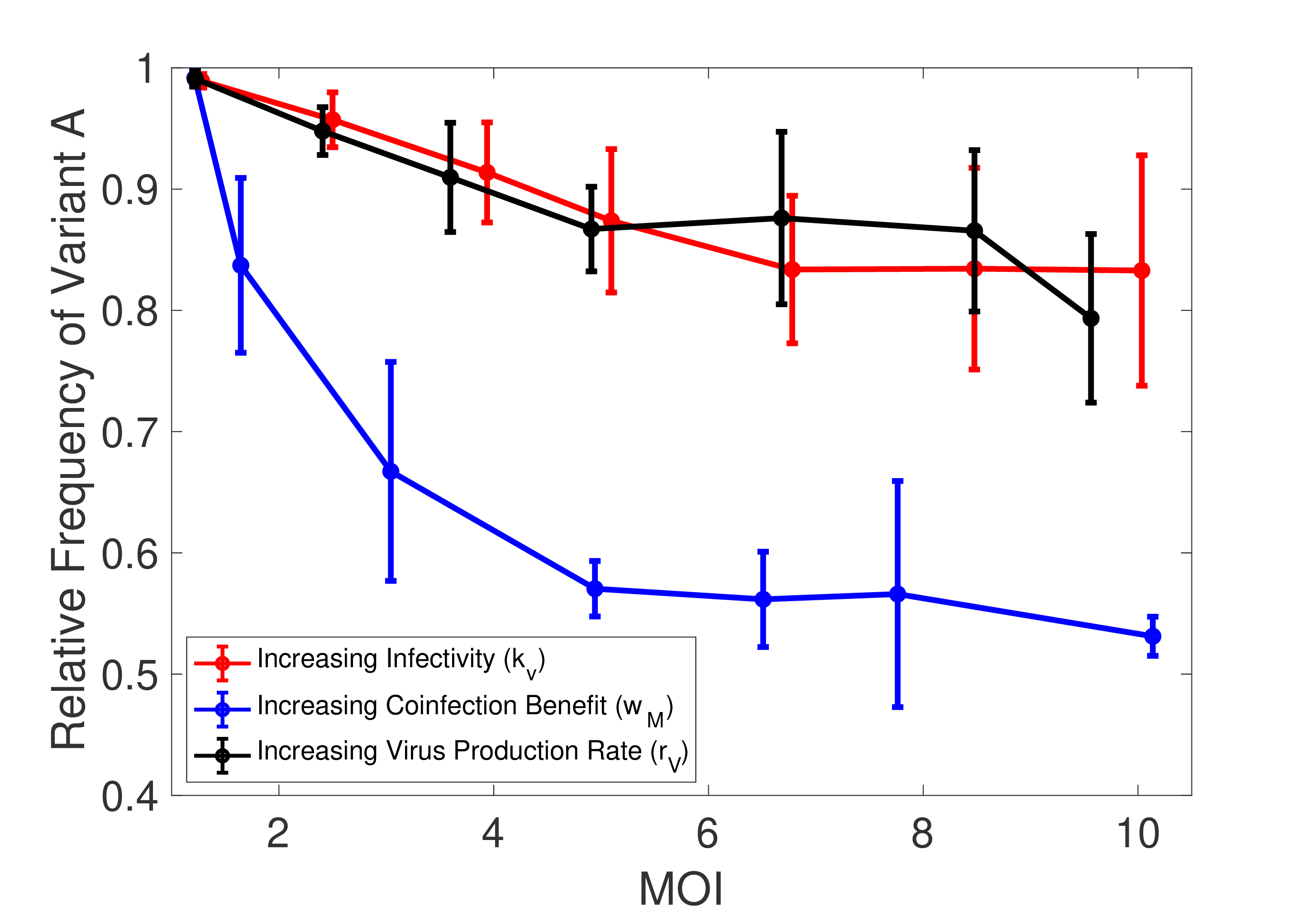

Supplement: Supplementary Data [file vey028_supp.zip › coinf_figs4.tiff]
